# MIP Models and Hybrid Algorithms for Simultaneous Job Splitting and Scheduling on Unrelated Parallel Machines

**DOI:** 10.1155/2014/519520

**Published:** 2014-02-26

**Authors:** Duygu Yilmaz Eroglu, H. Cenk Ozmutlu

**Affiliations:** Department of Industrial Engineering, Uludag University, Gorukle Campus, 16059 Bursa, Turkey

## Abstract

We developed mixed integer programming (MIP) models and hybrid genetic-local search algorithms for the scheduling problem of unrelated parallel machines with job sequence and machine-dependent setup times and with job splitting property. The first contribution of this paper is to introduce novel algorithms which make splitting and scheduling simultaneously with variable number of subjobs. We proposed simple chromosome structure which is constituted by random key numbers in hybrid genetic-local search algorithm (GAspLA). Random key numbers are used frequently in genetic algorithms, but it creates additional difficulty when hybrid factors in local search are implemented. We developed algorithms that satisfy the adaptation of results of local search into the genetic algorithms with minimum relocation operation of genes' random key numbers. This is the second contribution of the paper. The third contribution of this paper is three developed new MIP models which are making splitting and scheduling simultaneously. The fourth contribution of this paper is implementation of the GAspLAMIP. This implementation let us verify the optimality of GAspLA for the studied combinations. The proposed methods are tested on a set of problems taken from the literature and the results validate the effectiveness of the proposed algorithms.

## 1. Introduction

This study focuses on the problem of scheduling jobs that involve splitting and machine- and sequence-dependent setup times on nonidentical (unrelated) parallel machines to minimize the maximum completion time (makespan). The problem will be referred to as *R*_*m*_/*S*_*ijk*_/*C*_max⁡_ with job splitting.

Job splitting is a great requirement for some industries. For example, drilling of printed circuit board (PCB) manufacturing, dicing of semiconductor wafer manufacturing, and weaving of textile manufacturing are major bottleneck operations and splitting the jobs into subjobs and processing them on different machines are necessary because of the due date pressure and competitive challenges of the modern manufacturing industry. In fact, the methods that were proposed in this paper can be applied to real weaving process of textile manufacturing industry from which this research is originated.

The mentioned problem has a set of *n* jobs (*N* = {1, …, *n*}), which can be split into subjobs. The number of subjobs for each job will be between one and the maximum number of subjobs. The maximum number of subjobs will be defined beforehand and the proposed algorithms will try to find the best number of subjobs for each job. More setup times would be required if there are more subjobs. Hence, in the scheduling problem considered in this study, finding an appropriate number of subjobs to be split from each job is so important.

If the jobs' processing times are dependent on the assigned machines and there is no relationship among these machines, then the machines are considered to be unrelated [1]. So, the processing time of Job_*j*_ is different from Job_*k*_ on Machine_*i*_ in unrelated machine environment. As it will be explained in next sections, *p*_*ij*_ indicates processing time of Job_*j*_, at Machine_*i*_. The mentioned subjobs must be processed on one machine in a set *M* = {1, …, *m*} of *m* unrelated parallel machines (*R*_*m*_). The setup times are sequence- and machine-dependent (*S*_*ijk*_). Each machine has its own matrix of setup times, and these matrices are different from each other. The setup time on Machine_*i*_ between Job_*j*_ and Job_*k*_ is different compared with the setup time between Job_*k*_ and Job_*j*_ on the same machine.

In scheduling theory, the makespan (*C*_max⁡_) is defined as the completion time of the final job (when it leaves the system). A smaller *C*_max⁡_ implies a higher utilization, which is closely related to the throughput rate of the system. Therefore, reducing *C*_max⁡_ will lead to a higher throughput rate [2]. For that reason, minimization of the “makespan” is the objective of this study.

Minimizing the makespan on a scheduling problem with *m* identical parallel machines and sequence-dependent setup times is NP-hard [3]. Thus, a more complex case of the problem with job splitting and unrelated parallel machines is also NP-hard.

The present paper contains mixed integer programming (MIP) models and hybrid genetic-local search algorithm techniques to solve the mentioned *R*_*m*_/*S*_*ijk*_/*C*_max⁡_ with job splitting problem. The proposed algorithms in this study perform job splitting and scheduling simultaneously with variable number of subjobs, where, to the best of our knowledge, no work has been published on an algorithm with these properties. This is the first contribution of this paper. The second contribution of this work is getting over the problem of main difficulty in using random key numbers in the chromosome for hybrid structures. We proposed simple chromosome structure which is constituted by random key numbers in hybrid genetic-local search algorithm (GAspLA). In the literature random key numbers are frequently used in chromosome. But local search application inside the genetic algorithm made the situation more complex. Inside each generation of the genetic algorithm, we are trying to find better job sequence using local search. But the better job sequence must be adapted to the chromosome which will be used in each next generation. We need to determine which data in genes will be exchanged or in which condition and in which genes we will regenerate new random key numbers. To manage the random key numbers according to the desired job sequence that would be necessary during local search operations, which is implemented in the genetic algorithm, we developed algorithms that satisfy the adaptation of results of local search into the genetic algorithms with minimum relocation operation of genes' random key numbers. The third contribution of this paper is three developed mixed integer programming (MIP) models which are designed to solve different subjob environments. In the MIP1 and the MIP2, numbers of the subjobs will be determined by models between one and the maximum number of subjob. In the MIP3, the numbers of subjobs will be determined randomly between one and maximum number of subjobs beforehand and will be given as an input data to the model. In the MIP1, the quantities of subjobs are not equal. In the MIP2 and MIP3 the quantities of subjobs are equal. Finally we presented an implementation (GAspLAMIP) which is the fourth contribution of this study. In this implementation, obtained scheduling using GAspLA is combined with the MIP formulation. So, the result of the GAspLA feeds MIP formulation with initial solution set. This implementation lets us verify the optimality of GAspLA for the studied combinations. To present the performance of the algorithms, the problem set *SchedulingResearch* 2005 [4] was used. The results demonstrated that proposed algorithms outperformed the compared ones.

This paper is organized as follows.  Section 2 provides a review of the existing literature. In Section 3, mixed integer programming (MIP) mathematical models are formulated. In Section 4, the proposed GAspLA is described. In Section 5, the implementation of GAspLAMIP is presented.In Section 6, the experimental design and computational results are reported.  Section 7 concludes the paper.

## 2. Literature Review

The interest in scheduling problems with setup times began in the mid-1960s. These types of problems have received continuous interest from researchers since then. Allahverdi et al. [5] presented a survey of scheduling problems with setup times or costs. The paper classified scheduling problems into those with batching and nonbatching considerations and those with sequence-independent and sequence-dependent setup times. It also classified the literature according to shop environments, including a single machine, parallel machines, flow shop, no-wait flow shop, flexible flow shop, job shop, and open shop. Machine setup time is a significant factor for production scheduling in manufacturing environments, and sequence-dependent setup times have been investigated by researchers. Zhu and Wilhelm [6] presented a review of scheduling problems that involves sequence-dependent setup times (costs). Li and Yang [7] presented a review on nonidentical parallel-machine scheduling research in which the total weighted completion times are minimized. Models and relaxations are classified in this paper, and heuristics and optimizing techniques are surveyed for the problems. Lin et al. [8] studied research that compares the performance of various heuristics for unrelated parallel machine scheduling problems. They proposed a metaheuristic, and computational results showed that the proposed metaheuristic outperformed other existing heuristics for each of the three objectives, minimized makespan, total weighted completion time, and total weighted tardiness, when run with a parameter setting that is appropriate for the objective.

Because of the problem complexity, it is general practice to find an appropriate heuristic rather than an optimal solution for the parallel-machine scheduling problem. Park et al. [9] used a neural network and heuristic rules to schedule jobs with sequence-dependent setup times on parallel machines. To calculate the priority index of each job, they utilized a neural network, and their computational results showed that the proposed approach outperformed the Lee et al. [10] original ATCS (Apparent Tardiness Cost with Setups) and a simple application of ATCS. van Hop and Nagarur [11] focused on scheduling problems of *n* printed circuit boards (PCBs) for *m* nonidentical parallel machines, and a composite genetic algorithm was developed to solve this multiobjective problem. Test results of the proposed methodology showed that the solutions were efficient and were obtained within a reasonable amount of time. Zandieh et al. [12] studied on the hybrid flow shop scheduling problems in which there were sequence-dependent setup times. They proposed an immune evolutionary algorithm (IEA) for this problem. In the paper of Wang [13] the problem of single machine common due date scheduling with controllable processing times is considered. Behnamian et al. [14] compared makespan results solved by ant colony optimization (ACO), variable neighborhood search (VNS), simulated annealing (SA), and the VNS hybrid algorithm for the problem of parallel machine scheduling problems with sequence-dependent setup times. Yang [15] proposed an evolutionary simulation optimization approach for solving the parallel-machine scheduling problem. The proposed methodology's findings were benchmarked against lower-bound solutions, and efficient results were obtained. Balin [16] attempted to adapt a GA to the nonidentical parallel machine scheduling problem and proposed an algorithm with a new crossover operator and a new optimality criterion. The new algorithm was tested on a numerical example by implementing it in simulation software. The results showed that, in addition to its high computational speed for a larger-scale problem, the GA addressed the nonidentical parallel machine scheduling problem of minimizing the makespan. Keskinturk et al. [17] aimed to minimize the average relative percentage of imbalance and used the ACO algorithm for load balancing in parallel machines with sequence-dependent setup times. The results of tests on various random data showed that the ACO outperformed the GA and heuristics.

In this section, some of the previous studies on the unrelated parallel machine scheduling problem with the objective of minimizing the makespan are discussed. All of these studies considered in the following assumptions:

- machine-dependent and job sequence-dependent setup times;
- all of the jobs are available at time zero.

A metaheuristic Meta-RaPS was introduced by Rabadi et al. [18], and its performance was evaluated by comparing its solutions to those obtained by existing heuristics. The results of the metaheuristic showed that the solutions were efficient. A two-stage ant colony optimization (ACO) algorithm was proposed by Arnaout et al. [19]. The performance of this metaheuristic was evaluated by using the benchmark problems, and the solutions were found to be efficient. Another method was suggested by Chang and Chen [20] for the same NP-hard problem. A set of dominance properties was developed, including intermachine and intramachine switching properties, which are necessary conditions of job sequencing orders in a derived optimal schedule. They also introduced a new metaheuristic by integrating the dominance properties with a genetic algorithm (GADP). The performance of this metaheuristic was evaluated by using benchmark problems from the literature, and the solutions were efficient. Vallada and Ruiz [1] proposed a genetic algorithm that includes the crossover operator with a limited local search as well as a fast local search procedure; this method was tested on both small and large problem sets and outperformed the other evaluated methods. *SchedulingResearch* 2005 [4] datasets were used by many of the researchers [18–20] in the literature. Yilmaz Eroglu et al. [21] proposed a genetic algorithm with local search (GALA) that was based on random keys. To present the performance of the algorithm, the same problem set *SchedulingResearch* 2005 [4] was used. The results showed that the GALA, which is the foundation of this study, outperformed the other algorithms.

The jobs that are considered in this paper can be split into subjobs; a feature that is very seldom studied in the literature. Studies can be categorized as splitting the job into subjobs of discrete units or continuous units. Some studies that involve discrete units are as follows: Kim et al. [22] focused on the dicing of semiconductor wafer manufacturing, which is the major bottleneck operation of the whole process. They proposed a simulated annealing algorithm for the problem of allotting work parts of *L* jobs into *M* parallel unrelated machines, where a job is referred to a lot composed of *N* items. Setup times were job sequence-dependent. The proposed SA method outperformed a neighborhood search method in terms of the total tardiness. Kim et al. [23] suggested a two-phase heuristic algorithm for the problem of scheduling a drilling process in the PCB manufacturing system. It was assumed that a job can be split into a discrete number of subjobs and that they would be processed on identical parallel machines independently. In the first phase of the algorithm, an initial sequence is generated by existing heuristic methods for the parallel machine scheduling problem. In the second phase, each job was split into subjobs, and then jobs and subjobs were rescheduled on the machines by using a certain method. The performance of the suggested algorithm was proved by the results of the computational experiments, which performed better than an existing method. Shim and Kim [24] also focused on PCB manufacturing system bottleneck operations of the drilling process. For the problem of scheduling jobs that can be split into subjobs, they developed several dominance properties and lower bounds and then suggested a branch and bound algorithm using them. The suggested algorithm solved problems of moderate size in a reasonable amount of computational time. Xing and Zhang [25] proposed a heuristic algorithm for the *P*/split/*C*_max⁡_ problem and analyzed the worst case performance of the algorithm. Yalaoui and Chu [3] considered a simplified real-life identical parallel machine scheduling problem with sequence-dependent setup times and job splitting to minimize the makespan. The proposed method composed of two phases. In the first phase, the problem was reduced to a single machine scheduling problem and was transformed into a traveling salesman problem (TSP), which could efficiently be solved by using Little's method. In the second phase, a derived initial solution was improved in a step-by-step manner, accounting for the setup times and job splitting. Tahar et al. [26] proposed a new method, which is an improvement of the method proposed by Yalaoui and Chu [3]. For the problem of splitting the job into continuous units, Serafini [27] studied the scheduling problem of looms in the textile industry. Jobs might be independently split over several specified machines, and preemption was allowed. It was assumed that there were uniform parallel machines and that there were no setup times. For the mentioned problem, heuristic algorithms were proposed for the objective of minimizing the maximum weighted tardiness. Yilmaz Eroglu et al. [28] proposed a genetic algorithm (without local search) that decided number of subjobs for each order and makes schedule simultaneously. Result of computations showed that the suggested algorithm could find solutions for problems with 75 machines and 111 jobs in a reasonable amount of CPU time. According to the results, the proposed GA outperformed existing system in makespan. Pimentel et al. [29] focused on the problem which is related with the knitting production process. MIP model was formulated and the heuristics and local search algorithms were proposed for the identical parallel machine scheduling problem with job splitting to minimize total tardiness.

## 3. MIP Mathematical Models

In this section, mixed integer programming (MIP) mathematical models are formulated to find optimal solutions for the unrelated parallel machine scheduling problem with sequence dependent setup times and job splitting. Three mathematical models are developed on this topic. In the MIP1 and the MIP2, numbers of the subjobs will be determined by models between one and the maximum number of subjob. In the MIP3, the numbers of subjobs will be determined randomly between one and maximum number of subjobs beforehand and will be given as an input data to the model. In the MIP1, the quantities of subjobs are not equal. In the MIP2 and MIP3 the quantities of subjobs are equal.

Mentioned problem has a set of *n* jobs (*N* = {1, …, *n*}) which can be split into subjobs. The numbers that are declared in a set of *B* = {*B*[1], …, *B*[*n*]} have different meaning in MIP1, MIP2, and MIP3.

The numbers in set *B* for MIP1 and MIP2 denote the maximum number of subjobs for each job. MIP1 and MIP2 will decide the number of subjobs between one and maximum number of subjobs which is defined in set of *B*. So, in MIP1 and MIP2 the numbers of subjobs are variable.

The numbers in set *B* for MIP3 will be predefined randomly between one and the maximum number of subjobs for each job. So, in MIP3 the numbers of subjobs are constant. But this constant number will be generated randomly between one and the maximum number of subjobs in order to approximate the hybrid genetic-local search algorithm which will be described in Section 4.

Mentioned subjobs have to be processed on one machine in a set *M* = {1, …, *m*} of *m* unrelated parallel machines (*R*_*m*_):

- *p*_*ij*_: processing time of Job_*j*_, *j* ∈ *N* at Machine_*i*_, *i* ∈ *M*.
- *S*_*ijk*_: machine based sequence dependent setup time on Machine_*i*_, *i* ∈ *M*, when processing Job_*k*_, *k* ∈ *N*, after having processed Job_*j*_, *j* ∈ *N*.

Details about the models are explained in the following subsections.

### 3.1. MIP1: The Quantities of Subjobs Are Not Equal

The quantities of subjobs are not equal in MIP1. This is the point of difference of this model from hybrid genetic-local search algorithm (GAspLA) that will be explained in Section 4:

- *q* ^*p*^: total constant production quantity for each job.
- *D*_*ij*_ = *p*_*ij*_/*q* ^*p*^: processing time of unit quantity of Job_*j*_ at Machine_*i*_, *i* ∈ *M*.

The decision variables for the model are as follows:

(1) $$\begin{array}{c} X_{ijk}=\{\begin{array}{cc} 1, & {\text{if}\;{\text{Job}}_{j}\;\text{precedes}\;{\text{Job}}_{k}\;\mathrm{on}\;\text{Machine}}_{i}^{\;}\\ 0, & \text{otherwise,} \end{array}\\ Q_{ij}=\text{Quantity}\;\text{of}\;{\text{Job}}_{j}\;\text{that}\;\text{processed}\;\mathrm{on}{\;\text{Machine}}_{i}^{\;},\\ C_{ij}=\text{Completion}\;\text{time}\;\text{of}\;{\text{Job}}_{j}\;\text{at}{\;\text{Machine}}_{i}^{\;},\\ C_{\max}=\text{Maximum}\;\text{completion}\;\text{time.} \end{array}$$

The objective function is

(2) $$\begin{array}{c} {\text{Minimize}\;C}_{\max}. \end{array}$$

And the constraints are

(3) ∑i∈M∑ j∈{0}∪{N}j≠kXijk≤B[k],   ∀k∈N,

(4) ∑i∈M∑ j∈{0}∪{N}j≠kXijk≥1, ∀k∈N,

(5) Qik≤V∗∑j∈{0}∪{N}j≠kXijk, ∀i∈M,  ∀k∈N,

(6) $$\begin{array}{c} \sum_{i\in M}^{}Q_{ij}=q^{p},\;\forall j\in N, \end{array}$$

(7) $$\begin{array}{c} \sum_{i\in M}^{}Q_{i0}=0, \end{array}$$

(8) ∑k∈Nj≠kXijk≤1, ∀j∈N,  ∀i∈M,

(9) $$\begin{array}{c} \sum_{k\in N}^{}X_{i0k}\leq 1,\;\forall i\in M, \end{array}$$

(10) ∑h∈{0}∪{N}h≠k,h≠jXihj≥Xijk, ∀j,k∈N,  j≠k,  ∀i∈M,

(11) Cik+V∗(1−Xijk)≥Cij+Sijk+Dik∗Qik,∀j∈{0}∪{N}, ∀k∈N, j≠k, ∀i∈M,

(12) $$\begin{array}{c} C_{i0}=0,\;\forall i\in M, \end{array}$$

(13) $$\begin{array}{c} C_{ij}\geq 0,\;\forall j\in N,\;\;\forall i\in M, \end{array}$$

(14) $$\begin{array}{c} C_{\max}\geq C_{ij},\;\forall j\in N,\;\;i\in M, \end{array}$$

(15) Xijk∈{0,1}, ∀j∈{0}∪{N},  k∈N,  j≠k,  ∀i∈M

(16) $$\begin{array}{c} Q_{ij\;}\geq 0\;\text{and}\;\text{Integer},\;\forall j\in N,\;\;\forall i\in M. \end{array}$$

The objective (2) is to minimize the maximum completion time or makespan. Constraints sets (3) and (4) ensure that the number of subjobs will be between 1 and the maximum number of related subjobs. The usage of dummy jobs 0 as *X*_*i*0*k*_ indicates that *k* is the first job on Machine_*i*_. Constraint set (5) is to control the production quantities. If Job_*k*_ is processing on Machine_*i*_ then production quantity *Q*_*ik*_ exists; otherwise, production quantity is zero. Constraint set (6) ensures that the total subjob quantity of the each job is equal to a constant number *q* ^*p*^. Difference between unit processing times of each job will be calculated by the formulation of *D*_*ij*_ = *p*_*ij*_/*q* ^*p*^ which will be used in constraint set (11). Constraints set (7) defines total production quantities as 0 for dummy jobs. Constraints set (8) prevents subjobs on the same machine. Constraints (9) specify that not more than one subjob can be scheduled first at each machine. With set (10) we ensure that jobs are properly linked in machine: if a given Job_*j*_ is processed on a given Machine_*i*_, a predecessor *h* must exist on the same machine. Constraint set (11) is to control the completion times of the jobs at the machines. If Job_*k*_ is assigned to Machine_*i*_ after Job_*j*_, *X*_*ijk*_ will be equal to 1. In this situation the completion time of *k*, *C*_*ik*_ must be greater than the completion time of *j*, *C*_*ij*_ plus the setup time between *j* and *k* and processing time of *k*. The processing time of *k* is computed by multiplying unit processing time *D*_*ik*_ and *Q*_*ik*_ which is production quantity of Job_*j*_ on Machine_*i*_. If *X*_*ijk*_ is equal to 0, then the big constant *V* renders the constraint redundant. Sets (12) and (13) define completion times as 0 for dummy jobs and nonnegative for regular jobs, respectively. Set (14) defines the maximum completion time. Set (15) defines binary variables. Finally, set (16) defines integer variables.

Optimal solution for the problem can be obtained by solving this MIP model using a solver. We coded this model using MPL and CPLEX 11.0 is used as a solver. The model can solve this problem for 2-machine 6-job problem. In which all the jobs can be split into maximum 3 subjobs. But, for the 4-machine 6-job problem the model cannot give the optimal solution in reasonable elapsed time, one day. Because of this situation, additional constraints are added to this model to reduce the solution space. The new model, MIP2, that will be explained in Section 3.2 is identical with the GAspLA that will be explained in Section 4.

### 3.2. MIP2: The Quantities of Subjobs Are Equal

In this model, each subjob from one main job includes equal quantity of order. The model is also deciding the optimal number of subjobs. So, the content of this model is identical with the GAspLA that will be explained in Section 4.

To achieve equal subjob quantities in MIP2, constraint sets (17) and (18) will be added to the MIP1 that was explained in the previous subsection. Following logic constructs the constraints sets (17) and (18):

- If there is a splitting operation, the production quantities of Job_*j*_ on Machine_*i*_ and Machine_*t*_ must be equal (*Q*_*ij*_ = *Q*_*tj*_).
- If there is splitting, then |*Q*_*ij*_ − *Q*_*tj*_| = 0 must be satisfied.
- Splitting condition is added to the inequality of |*Q*_*ij*_ − *Q*_*tj*_| ≤ *ε*.

Consider

(17) Qij−Qtj≤0.1+V∗(2−∑h∈{0}∪{N}h≠jXihj−∑h∈{0}∪{N}h≠jXthj  )∀i,t∈M, i≠t, ∀j∈N,

(18) Qij−Qtj≥−0.1−V∗(2−∑h∈{0}∪{N}h≠jXihj−∑h∈{0}∪{N}h≠jXthj)∀i,t∈M, i≠t, ∀j∈N.

The quantities of subjobs (*Q*_*ij*_  and *Q*_*tj*_) are integer, because of this constraint, *ε* or 0.1 difference between subjobs will lose the importance and the constraint will be provided. Otherwise, the big constant *V* renders the constraint redundant.

We coded this model using MPL and CPLEX 11.0 is used to solve this model. While the model can solve the problem with 2 machines and 6 jobs, the 4-machine 6-job problem could not be solved even in one day. This circumstance has forced us to develop new model, MIP3, that is explained in Section 3.3.

### 3.3. MIP3: The Quantities of Subjobs Are Determined

In MIP3, the numbers of subjobs are determined before the model start running. To become close to the GAspLA, the numbers of subjobs are set randomly between 1 and the maximum number of subjobs for each individual jobs. The details about the model are as follows.

The decision variables for the model are as follows:

(19) $$\begin{array}{c} X_{ijrks}=\{\begin{array}{cc} 1, & \text{if}\;\;jr\;\text{which}\;\text{is}\;r\text{th}.\;\text{sub}\;\text{job}\;of\;{\text{Job}}_{j}\;\text{is}\\ & \text{precedes }ks\;\text{which is}\;s\text{th}.\;\text{sub}\;\text{job}\;\text{of}\\ & {\text{Job}}_{k}\mathrm{on}\;{\text{Machine}}_{i}\\ 0, & \text{otherwise,} \end{array}\\ C_{ijr}={\text{Completion}\;\text{time}\;\text{of}\;\text{sub job}\;jr\;\mathrm{on}\;\text{Machine}}_{i},\\ C_{\max}=\text{Maximum}\;\text{Completion}\;\text{Time.} \end{array}$$

The objective function is

(20) $$\begin{array}{c} {\text{Minimize}\;C}_{\max}. \end{array}$$

The constraints are

(21) $$\begin{array}{c} \sum_{i\in M}^{}\sum_{\;j\in \left\{0\right\}\cup \left\{N\right\}}^{}\sum_{\;r\in B}^{}X_{ijrks}=1,\;\forall k\in N,\;\;\forall s\leq B\left[k\right], \end{array}$$

(22) $$\begin{array}{c} \sum_{\;i\in M}^{}\sum_{\;k\in N}^{}\sum_{\;s\in B}^{}X_{ijrks}\leq 1,\;\forall j\in N,\;\;\forall r\leq B\left[j\right], \end{array}$$

(23) $$\begin{array}{c} \sum_{\;r\in B}^{}\sum_{\;k\in N}^{}\sum_{\;s\in B}^{}X_{i0rks}\leq 1,\;\forall i\in M, \end{array}$$

(24) ∑h∈{0}∪{N}h=j⇒m≠rh=k⇒m≠s∑ m∈BXihmjr≥Xijrks∀i∈M, ∀j∈N, ∀r≤B[j],∀k∈N, ∀s≤B[k]

(25) Ciks+V(1−Xijrks)≥Cijr+Sijk+pikB[k]∀i∈M, ∀j∈{0}∪{N}, ∀r≤B[j],∀k∈N, ∀s≤B[k]

(26) $$\begin{array}{c} C_{i0r}=0\;\forall i\in M,\;\;\forall r\leq B\left[j\right] \end{array}$$

(27) $$\begin{array}{c} C_{ijr}\geq 0\;\forall i\in M,\;\;\forall j\in N,\;\;\forall r\leq B\left[j\right] \end{array}$$

(28) $$\begin{array}{c} C_{\max}\geq C_{ijr}\;\forall i\in M,\;\;\forall j\in N,\;\;\forall r\leq B\left[j\right] \end{array}$$

(29) Xijrks∈{0,1}∀j∈{0}∪{N}, ∀r≤B[j],∀k∈N, ∀s≤B[k],∀i∈M, j=k⟹r≠s.

The objective (20) is to minimize the maximum completion time (makespan). Constraint set (21) ensures that every subjob is assigned to one machine and has one predecessor. The usage of dummy job 0 as *X*_*i*0*rks*_ indicates that *ks*, which is *s*th subjob of Job_*k*_, is the first job on Machine_*i*_. Constraints (22) make sure that the maximum number of successors of every subjob is one. Constraints (23) specify that not more than one subjob can be scheduled first at each machine. Constraint set (24) ensures that jobs are properly linked in machine. If subjob *jr* is processed on a given Machine_*i*_, a predecessor *hm* must be processed on the same machine. Constraints (25) are used to calculate and control completion times. If a subjob *ks* is assigned to Machine_*i*_ after subjob *jr*, then *X*_*ijrks*_ = 1. In that condition *C*_*iks*_ must be greater than or equal to the completion time of *jr* and *C*_*ijr*_ plus the setup time between Job_*j*_ and Job_*k*_ and the processing time of subjob *ks* which is calculated by processing time of Job_*k*_ on Machine_*i*_, *p*_*ik*_, divided by the number of subjobs of Job_*k*_, *B*[*k*]. If *X*_*ijrks*_ = 0, then the big constant *V* renders the constraint redundant. Constraints (26) state that the completion time for the dummy job 0 is zero and constraints (27) ensure that completion times are nonnegative for regular jobs. Set (28) defines the maximum completion time. Set (29) defines the binary variables.

MIP3's performance is better than the others and gave optimal solution to many of the problem sets which will be explained in the computational results. We coded this model using MPL and to solve the models, CPLEX 11.0 is used as a solver.

## 4. GAspLA: Hybrid Genetic-Local Search Algorithm for Job Splitting Property

### 4.1. Genetic Algorithm for Job Splitting Property

The genetic algorithm (GA) is a search technique based on the principles of genetics and natural selection. One of the important issues is the genetic representation (string of symbols) of each solution in a population. The string is referred to as chromosome and the symbols as genes. After generation of initial population and determination of the fitness function values for each chromosome, the GA manipulates the selection process by operations such as reproduction, crossover, and mutation. The algorithm was introduced in the 1970s by Holland [30]. As the searching technique of genetic algorithms (GAs) [31] became popular in the mid-1980s, many researchers started to apply this heuristic to scheduling problems.

The encoding must be designed to utilize the algorithm's ability to transfer information among chromosome strings efficiently and effectively [31]. To qualify our encoding scheme, the permutation of jobs in this work is shown through random keys. Each job has a random number between 0 and 1. These random keys show the relative order of the jobs for each machine. In addition, each chromosome also carries the number of subjobs. Detailed description of developed genetic algorithm has been reported in the following subsections.

The proposed local search algorithm which will be explained in Section 4.2 is inserted in fitness function calculation process of genetic algorithm. The proposed hybrid genetic-local search algorithms are coded in C# language according to proposed methods that were explained in the next subsections.

#### 4.1.1. Encoding Scheme

**Figure 1:**
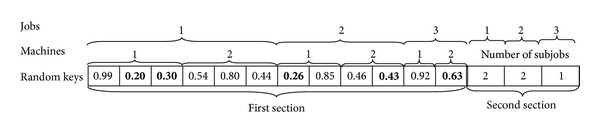
Representation of a chromosome in the GA.

A chromosome is presented as a string of random keys.  Figure 1 illustrates a sample chromosome. We would like to note that this chromosome structure is used in our previous study [28] which proposed a genetic algorithm without local search. In the first section of the chromosome, the string contains *n* (number of jobs) sections. Each section is further divided into *m* (number of machines) sections. Each section for machines is divided into subsections (genes). The number of subsections for related machine is determined according to maximum number of subjobs for related job. Sequence of jobs will be determined by random key numbers (generated between 0 and 1) of each gene. In the second section, the string contains *n* (number of jobs) sections. For each section, a random number will be generated between one and the max numbers of subjobs for each job to determine the number of subjobs. The chromosome structure of the example is in Figure 1. For this chromosome structure, we have 2 machines and 3 jobs. Maximum numbers of subjobs for Job_1_, Job_2_, and Job_3_ are 3, 2, and 1, respectively. For Job_1_, because of number of subjobs as the value of 2 in second section of chromosome, the smallest two random numbers will be selected from first section of the chromosome among all the numbers generated for Job_1_. Selected values (0.2 and 0.3 for Job_1_) are given in bold in Figure 1. It's clear that the first and second subjobs of Job_1_ will be processed on Machine_1_. Similarly, number of subjobs is 2 for Job_2_ and they will be processed on Machine_1_ and Machine_2_. Number of subjobs is 1 for Job_3_ and it will be processed on Machine_2_.

#### 4.1.2. Fitness Function (*C*_max⁡_)

As mentioned earlier, there are machine-dependent processing times and machine and job sequence-dependent setup times:

- *p*_*ij*_: processing time of Job_*j*_, *j* ∈ *N* at Machine_*i*_, *i* ∈ *M*.
- *S*_*ijk*_: machine based sequence dependent setup time on Machine_*i*_, *i* ∈ *M* when processing Job_*k*_ *k* ∈ *N* after having processed Job_*j*_, *j* ∈ *N*.

**Figure 2:**
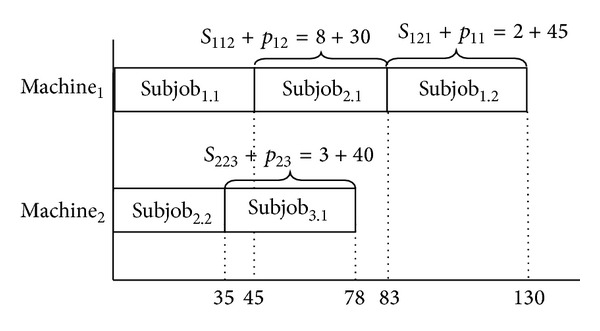
Resulting schedule and *C*_max⁡_ for chromosome in Figure 1.

In case of considering three jobs, two machines scheduling problem, for example, Table 1 is the processing times for Machine_1_ and Machine_2_. Tables 2 and 3 are setup times of Machine_1_ and Machine_2_, respectively, for the mentioned jobs. According to chromosome that was shown in Figure 1, Machine_1_ will process two subjobs of Job_1_ (Subjob_1.1_, Subjob_1.2_) and one subjob of Job_2_ (Subjob_2.1_). Machine_2_ will process one subjob of Job_2_ (Subjob_2.2_) and one subjob of Job_3_ (Subjob_3.1_). The sequence of jobs on Machine_1_ will be determined according to random key numbers (0.20, 0.26, and 0.30). Increasing arrangement of these random numbers will designate Machine_1_'s job sequence. So, sequence of subjobs is Subjob_1.1_, Subjob_2.1_ and Subjob_1.2_ on Machine_1_. If a job is split into subjobs, processing time of required subjob on selected machine can be calculated by dividing a job's processing time on the related machine to the number of subjobs. Completion time of Machine_1_ is 130 and completion time of Machine_2_ is 78 according to *p*_*ij*_ and *S*_*ijk*_ values. So, *C*_max⁡_ will be 130. Mentioned chromosome's schedule and makespan value can be seen in Figure 2. Population size will determine number of different chromosomes and *C*_max⁡_ values.

#### 4.1.3. Genetic Operators

After generating initial population, selection, crossover, and mutation will be iteratively used to search for the best solution.

**Figure 3:**
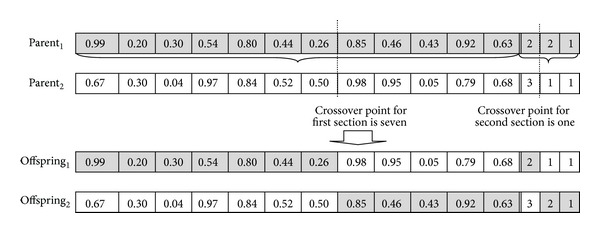
Crossover operation: two parents mate in order to produce two offsprings.

**Figure 4:**
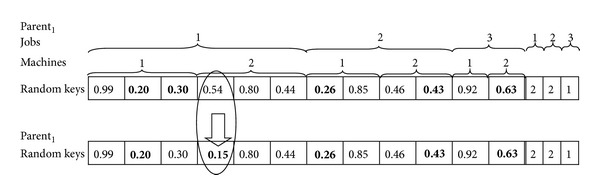
Mutation operation.

- Selection: chromosomes will be selected into the mating pool based on random selection method [32]. In this method, moms and dads are randomly chosen from population.
- Crossover: the crossover operator, which was applied according to value of crossover rate, *P*_*c*_, is a method for sharing information between chromosomes. Single point crossover will be used as crossover operator in our algorithm. It randomly chooses the crossing point and exchanges the genes between two parents in order to create offsprings. Crossover operator will be used for the first and second sections separately.  Figure 3 illustrates this operation. In Figure 3, for the first section the crossing point is selected randomly at position seven. The child is created as follows. First, Parent_1_ passes its genes that are on the left of the crossover point (position seven) to Offspring_1_. In a similar manner, Parent_2_ passes its genes to the left of the same crossover point to Offspring_2_. Next, the genes on the right of the crossover point of Parent_1_ (Parent_2_) are copied to Offspring_2_ (Offspring_1_). For the second section, crossing point is selected at position one randomly. Same procedure to create the offsprings is applied for the second section of the chromosome.
- Mutation: the mutation operator is used to prevent converging to a local optimum. In our algorithm, a mutation is performed as follows. Chromosomes that mutation operation will be applied to are randomly selected from the population according to mutation rate (*P*_*m*_). The value of the randomly selected gene of the chromosome will be replaced with a new random number. This operation will be applied to all selected chromosomes. In this way, chromosomes with new schedules and makespan values can be obtained. The fitness value may be better or worse or may not be changed after applying this operator. For the example in Figure 4 if randomly selected chromosome is Parent_1_ and randomly selected gene's key number 0.54 would be changed to the key number 0.15 which is also randomly generated for the mutation operation, the sequence on Machine_1_ and Machine_2_ will be altered. Because selected values for Job_1_ will be 0.20 and 0.15 (are given in bold in Figure 4), Machine_1_ will process one subjob of Job_1_ (Subjob_1.1_) and one subjob of Job_2_ (Subjob_2.1_). Machine_2_ will process one subjob of Job_1_ (Subjob_1.2_), one subjob of Job_2_ (Subjob_2.2_), and one subjob of Job_3_ (Subjob_3.1_). This will be a cause of new makespan value of 127.5 which is better than the previous one.

### 4.2. Local Search

Integrating one of the local search techniques within GA will generally generate more competitive results. The integrated dominance properties method was proposed by Chang and Chen [20]. In this method, the decision to exchange two jobs (intramachine or intermachines) is made according to the result of calculations, which are described in detail in the cited paper. While adapting this method to our GA, we designed a local search method based on integrated dominance properties. Our aim is searching all possible alternatives in chromosome in order to get better processing and setup times for each job. In the proposed local search method, the following three disparities were created as compared with integrated dominance properties method.

1. Job interchange: we only used setup times instead of adjusted processing times while interchanging two jobs that are processed on the same machine.
2. Job exchange: while the jobs under consideration are not on the same machine, setup times and processing times are both considered in the calculations.
3. During the job interchanges or exchanges on chromosome, also the random numbers must be redesigned. So, after the decision to interchange or exchange jobs is made, random numbers must be redesigned to achieve the required sequence. In order to change minimum number of random numbers on the chromosome, a new approach is generated. This process (calibration of random numbers) is also managed and coded in the proposed local search method.

All of the situations for local search have been considered and written in pseudocode, which is summarized in the Appendix. The proposed intermachine (interchange) and intramachine (exchange) job changes will be explained in Section 4.2.1 and the calibration of random numbers will be explained in Section 4.2.2. The notation for the pseudocode and explanations of local search are as follows.

*g*: number of selected genes from the chromosome, which is ordered by machine and then by job sequence. Each individual gene contains machine and job features.  Table 4 explains the sample gene structure for an example with seven jobs and two machines. The structure also indicates the sequence of jobs for each machine.

- *i* and *j*: comparing gene numbers to decide whether the jobs in these genes should be exchanged.
- Gene(*i*)_Machine_ indicates the machine number on *i*th gene. According to the structure shown in Table 4, the machine number for 1st gene (Gene(1)_Machine_) is 0.
- Gene(*i*)_job_ indicates the job number on *i*th gene. According to the structure shown in Table 4, the job number for 1st gene (Gene(1)_job_) is 4.
- ST_Mach(*i*)Job(*j*,*k*)_: setup time on Gene(*i*)_Machine_ between Gene(*j*)_job_ and Gene(*k*)_job_.
- PT_Mach(*i*),Job(*j*)_: processing time of Gene(*j*)_job_ on Gene(*i*)_Machine_
- CT_Gene(*i*)_Machine_: completion time of Gene(*i*)_Machine_

#### 4.2.1. Interchange and Exchange of Jobs

**Figure 5:**
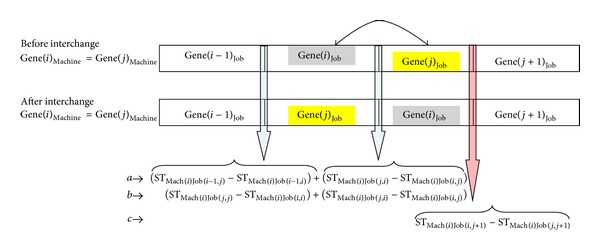
Intermachine interchange and adjacent jobs situation.

**Figure 6:**
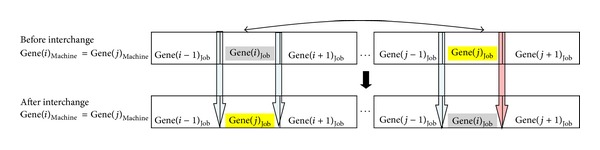
Intermachine interchange and nonadjacent jobs situation.

*(i) Interchange of jobs*: there are two cases to be considered within the intermachine interchange, they are adjacent jobs interchange (see Figure 5) and nonadjacent jobs interchange (see Figure 6).

*(I) Adjacent interchange*: in order to define the algorithm better, adjacent jobs interchange case is also demonstrated in Figure 5. Arrows on Figure 5 show setup time (among stated genes) differences between after interchange and before interchange situations. In Figure 5 and intermachine, adjacent jobs interchange section of pseudocode, steps are as follows:

1. if Gene(*i*−1)_job_ precedes Gene(*i*)_job_ on before interchange section of Figure 5, “*a*” represents sum of setup time differences between after interchange and before interchange situations for stated blue arrows.
2. If Gene(*i*)_job_ is the first job on the machine on before interchange section of Figure 5, “*b*” represents sum of setup time differences between after interchange and before interchange situations for stated blue arrows.
3. Either “*a*” or “*b*” must be equal to 0.
4. If Gene(*j*)_job_ precedes Gene(*j*+1)_job_ on Figure 5, “*c*” represents setup time differences between after interchange and before interchange situations for stated red arrow.
5. If the sum of *a*, *b*, and *c* is smaller than 0, it means that better completion time is obtained. Let us interchange Gene(*i*)_job_ and Gene(*j*)_job_ on the schedule.
6. Then calibrate random key numbers. In Section “4.2.2”, item “i” is description of intermachine interchange.

*(II) Nonadjacent interchange*: this case is shown in Figure 6. The steps to decide whether interchange of Gene(*i*)_job_ and Gene(*j*)_job_ on the schedule are similar with adjacent interchange. During the calculation of “*a*” and “*b*”, three blue arrows must be sum which are shown on Figure 6. This is caused by new setup time differences coming into existence between after interchange and before interchange situations. The remaining steps are the same as adjacent interchange situation.

**Figure 7:**
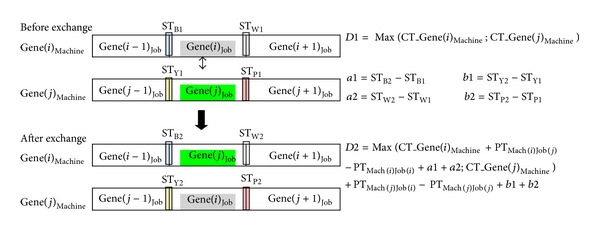
Intramachine exchange (exchange of Gene(*i*)_Job_ and Gene(*j*)_Job_ on the schedule).

*(ii) Intramachine exchanging is shown on Figure 7*: in Figure 7 and intramachine exchanging section of pseudocode steps are as follows:

1. calculate completion times of Gene(*i*)_Machine_ and Gene(*j*)_Machine_ before exchange. “*D*1” represents maximum value of these two values.
2. If Gene(*i*−1)_job_ precedes Gene(*i*)_job_ on Gene(*i*)_Machine_ which was shown before exchange section of Figure 7, “*a*1” represents setup time differences between after interchange and before interchange situations for stated blue rectangles (ST_B1_, ST_B2_). (*a*1 = ST_B2_ − ST_B1_.) (If Gene(*i*)_job_ is the first job on Gene(*i*)_Machine_, second “*a*1” of pseudocode represents sum of setup time differences between after interchange and before interchange situations.)
3. If Gene(*i*)_job_ precedes Gene(*i*+1)_job_ on Gene(*i*)_Machine_ which was shown before exchange section of Figure 7, “*a*2” represents setup time differences between after interchange and before interchange situations for stated white rectangles (ST_W1_, ST_W2_). (*a*2 = ST_W2_ − ST_W1_.)
4. If Gene(*j*−1)_job_ precedes Gene(*j*)_job_ on Gene(*j*)_Machine_ which was shown before exchange section of Figure 7, “*b*1” represents setup time differences between after interchange and before interchange situations for stated yellow rectangles (ST_Y1_, ST_Y2_). (*b*1 = ST_Y2_ − ST_Y1_.) (If Gene(*j*)_job_ is the first job on Gene(*j*)_Machine_, second “*b*1” of pseudocode represents sum of setup time differences between after interchange and before interchange situations.)
5. If Gene(*j*)_job_ precedes Gene(*j*+1)_job_ on Gene(*j*)_Machine_ which was shown before exchange section of Figure 7, “*b*2” represents setup time differences between after interchange and before interchange situations for stated pink rectangles. (ST_P1_, ST_P2_.) (*b*2 = ST_P2_ − ST_P1_.)
6. Calculate new completion times of Gene(*i*)_Machine_ and Gene(*j*)_Machine_ after possible exchange. “*D*2” represents maximum value of these two values. Formulation the computation of *D*2 is shown in Figure 7.
7. If *D*2 is smaller than *D*1 it means that better completion time is obtained. Let us exchange Gene(*i*)_job_ and Gene(*j*)_job_ on the schedule.
8. Then calibrate random key numbers. In Section “4.2.2”, item “ii” is description of intramachine exchange.

#### 4.2.2. Calibration of Random Numbers on the Chromosome

**Figure 8:**
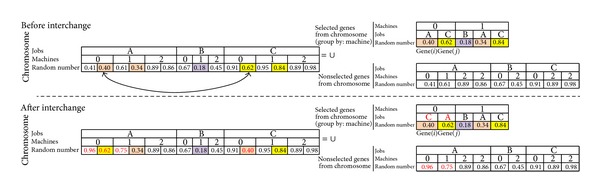
An example for calibration of random numbers on the chromosome for intermachine interchanges.

**Figure 9:**
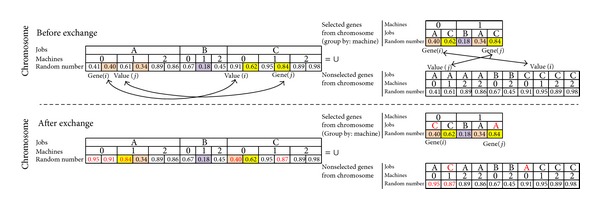
An example for calibration of random numbers on the chromosome for intramachine exchanges.

If we decided to change Gene(*i*)_job_ and Gene(*j*)_job_, we need to calibrate the chromosome to recognize the new situation. To recognize the job changes by chromosome, the following configurations were also integrated in the code. During the integration process, the places of random numbers are considered. Job_A_ is split into 2 subjobs, Job_B_ is not split, and Job_C_ is split into 2 subjobs for the considered chromosome. The number of subjobs has been determined randomly as pointed out before. Chromosomes of Figures 8 and 9 do not show the section of number of subjobs.

*(i) Intermachine interchange*: Figure 8 shows before and after interchange of jobs on the same machine. Selected genes from chromosome and nonselected genes from chromosome constitute a chromosome. The realized interchanges are shown in red letter on the after interchange section of Figure 8. The steps of interchanging Gene(*i*)_job_ and Gene(*j*)_job_ for the example of Figure 8 are the following.

- Random numbers of the jobs will be exchanged (0.40 for Gene(*i*)_job_ → Job_*A*_ and 0.62 for Gene(*j*)_job_ → Job_*C*_ will be changed to 0.40 for Gene(*i*)_job_ → Job_*C*_ and 0.62 for Gene(*j*)_job_ → Job_*A*_).
- 0.40 is smaller than 0.62. Thus, after the interchange there is no need to change the other random numbers for Job_C_. Because the current situation guarantees Job_C_ to select Machine_0_.
- 0.62 is bigger than 0.40. Thus, after the interchange of the other random numbers for Job_A_ those smaller than 0.62 inside the “nonselected genes from chromosome” must be changed. For example, 0.41 is smaller than 0.62. A new random number which is bigger than 0.62 will be randomly generated (0.96) and changed with 0.41. Another random number is 0.61 inside the “nonselected genes from chromosome” for Job_A_ which is smaller than 0.62. A new random number which is bigger than 0.62 will be randomly generated (0.75) and changed with 0.61. Thus, Job_A_ satisfies selecting Machine_0_.

*(ii) Intramachine exchange*: Figure 9 shows before and after exchange of jobs on two different machines. The realized exchanges are shown in red letter on the after exchange section of Figure 9. The steps of exchanging Gene(*i*)_job_ and Gene(*j*)_job_ for the example of Figure 9 are as follows. Value(*i*) is a maximum random number value for Gene(*j*)_job_ on Gene(*i*)_Machine_. Similarly, Value(*j*) is a maximum random number value for Gene(*i*)_job_ on Gene(*j*)_Machine_.

- Random numbers for Gene(*i*) and Value(*i*) are exchanged. And also random numbers for Gene(*j*) and Value(*j*) are exchanged.
- After the exchange it must be checked if there is a random number inside the “nonselected genes from chromosome” for Gene(*j*)_job_ → Job_*A*_ that is smaller than random number of Gene(*j*) which is equal to 0.84 or other selected value of Job_A_ which is equal to 0.34. For example, 0.41 is smaller than 0.84. Another random number which is bigger than 0.84 will be randomly generated (0.95) and changed with 0.41.
- Between selected genes from chromosome and nonselected genes from chromosome, jobs for Gene(*i*) and Value(*i*) are exchanged. And also jobs for Gene(*j*) and Value(*j*) are exchanged.
- It must be also checked if there is a random number inside the “nonselected genes from chromosome” for Gene(*i*)_job_ → Job_*C*_ that is smaller than random number of Gene(*i*) which is equal to 0.40 or other selected value of Job_C_ which is equal to 0.62. For example, 0.61 is smaller than 0.62. Another random number which is bigger than 0.62 will be randomly generated (0.87) and changed with 0.61.

## 5. Implementation: GAspLA-to-MIP Method (GAspLAMIP)

In this section, we propose GAspLA based mix-integer programming model for the mentioned problem. This implementation will help us to reduce the elapsed time of MIP3. And this implementation will also show that our hybrid genetic-local search algorithm (GAspLA) gives optimal results for the studied datasets which will be discussed in Section 6.

As we mentioned, we obtain final scheduling using GAspLA. Then, according to the sequencing information of subjobs on each machine, we set binary variable values in the MIP3 formulation as initial values. For example, according to Table 5, there are 4 machines and 6 jobs. Each machine's first job must be dummy job (*j* : 0, *r* : 1). According to this information, first row of Table 5 indicates that on Machine_1_, subjob_31_ will be processed. And according to second and third rows of Table 5, Machine_2_'s sequence will be subjob_11_ and subjob_41_. The schedules of the other machines can be interpreted similarly.

In order to reduce the search space of MIP3, the result schedule of GAspLA will be used. Therefore, running MIP3 with the initial solution of GAspLA will help us to accelerate to reach the solution. In Section 3.3, MIP3 was proposed and in Section 4 GAspLA was proposed. The steps for GAspLAMIP are as follows.

 
*Step *1. Run the GAspLA algorithm.
 
*Step *2. According to the final sequence information from GAspLA, specify the value of binary variable that MIP3 will use.
 
*Step *3. MIP3 will use these binary variables as initial solution.
 
*Step *4. Send the binary variable information to the MIP3 formulation.
 
*Step *5. Solve the MIP3 to find an optimal solution with given initial solution.

The results of the implementation will be discussed in the next section.

## 6. Experimental Design and Computational Results

The proposed algorithm is coded in C# language and run on computer with an Intel Core i7-3612QM processor running at 2.10 GHz. Test samples are provided by *SchedulingResearch* 2005 [4].

The parameter configuration of GALA that was proposed by Yilmaz Eroglu et al. [21], which is the foundation of GAspLA, is done by the Design of Experiment (DoE). The scheduling environment difference between GALA and GAspLA is job splitting property. We will make comparison also with GALA and to avoid advantages of parameter configuration, we run GALA, GAspLA, and GAspLAMIP with the same parameters which are obtained by DoE approach that is detailed in next subsection.

### 6.1. Experimental Results and Parameter Values

**Figure 10:**
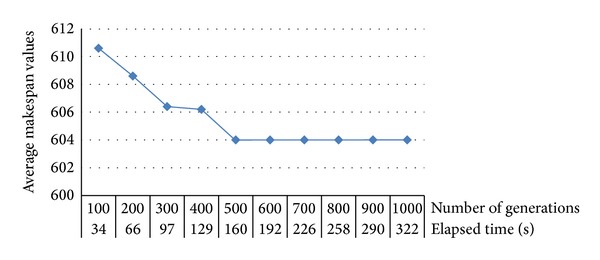
Average makespan values and elapsed times for the determined number of generations.

Makespan values and elapsed times were registered for the selected 4-machine 20-job problem structure for every 100 generations up to 1000 generations to determine the optimal number of generations.  Figure 10 provides the average makespan values and elapsed times of five replications for the analyzed generations with the selected parameters.  Figure 10 shows that after 500 generations, there is no improvement in the makespan. Thus, the number of generations can be set to 500.

**Figure 11:**
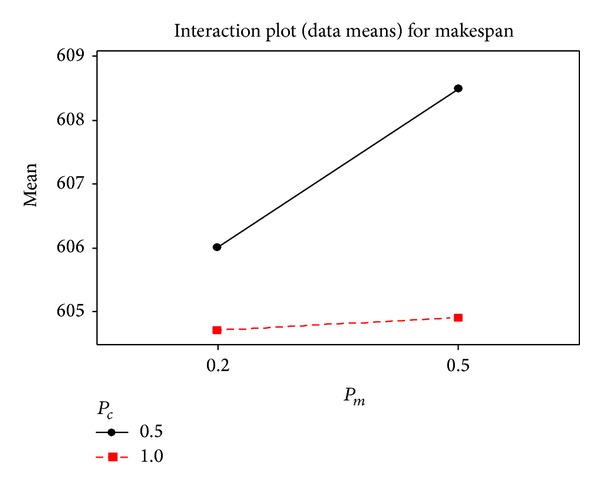
Interaction plot of *P*_*c*_ and *P*_*m*_.

**Figure 12:**
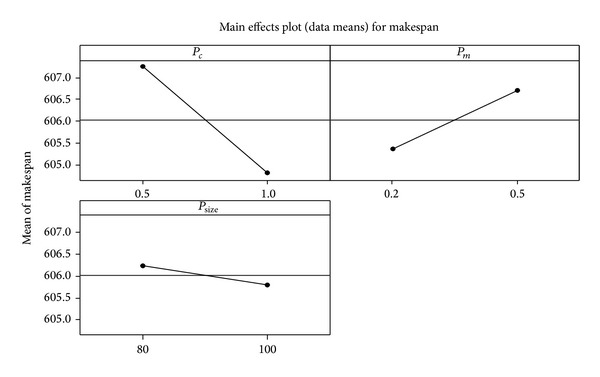
Main effects plot of the three factors.

The 4-machine 20-job problem structure was chosen to determine the optimal parameter setting by DoE. The levels of the three factors are listed in Table 6. Five replications were conducted for each combination, and the makespan values were calculated by running the program until 500 generations had elapsed. In Table 7, the estimates obtained through the regression analysis are shown, including significant factors (*P*-value ≤ 0.05). The results indicate that the probability of crossover (*P*_*c*_) and probability of mutation (*P*_*m*_) are significant, with low *P* values shown in Table 7. The interaction plot in Figure 11 and the low *P* value of *P*_*c*_∗*P*_*m*_ in Table 7 indicate that there is a significant interaction between *P*_*c*_ and *P*_*m*_. According to Figures 11 and 12, when *P*_*c*_ is set to 1 and *P*_*m*_ is set to 0.2, the algorithm will yield a better solution quality. The population size (*P*_size_) is set to 100 according to the main effects plot in Figure 12.

The obtained results are evaluated in two subsections. In the first subsection, the results of MIP that was proposed by Rabadi et al. [18] and the results of GALA which was proposed by Yilmaz Eroglu et al. [21] will be analyzed for nonjob splitting situation. For the job splitting situation, the results of MIP3 that was proposed in Section 3.3, the results of GAspLA that was proposed in Section 4, and the results of implementation (GAspLAMIP) that was proposed in Section 5 will be explicated in Section 6.2. In Section 6.3, detailed results of solving some literature problems using the proposed GAspLA are submitted.

### 6.2. The Results of MIP, GAspLA and GAspLAMIP

There are 15 different problem instances in the dataset *SchedulingResearch* 2005 that was taken from the literature [4]. For the combinations which were shown on the Table 8, the first instances of these datasets are used and the following cases are studied and reported.

- For the nonjob splitting situation, MIP model which was proposed by Rabadi et al. [18] is coded in MPL and solved using CPLEX 11.0. The makespan values and elapsed times are reported in the nonjob splitting situation, MIP0 section of Table 8.
- For the nonjob splitting situation, the makespan values and elapsed times of the GALA algorithm which was proposed by Yilmaz Eroglu et al. [21] are reported in the nonjob splitting situation, GALA section of Table 8.
- For the job splitting situation, the proposed model which is discussed in Section 3.3 was coded in MPL and solved using CPLEX 11.0. The makespan values and elapsed times are reported in job splitting situation, MIP3 section of Table 8.
- For the job splitting situation, the makespan values and elapsed times of the proposed GAspLA algorithm are reported in the job splitting situation, GAspLA section of Table 8.
- For the job splitting situation, the makespan values and elapsed times of the GAspLAMIP which is discussed in Section 5 are reported in job splitting situation, GAspLAMIP section of Table 8.

The results of the models whose elapsed times exceed one day (86400 seconds) are not reported in Table 8. To point out the improvement through the proposed algorithm for the problem with job splitting, nonjob splitting situation results are also reported. If the analyzed problem has the job splitting property, then it is better to use the proposed algorithms including job splitting. The results on Table 8 can be interpreted as follows.

For the nonjob splitting situation, MIP0 model that was proposed by Rabadi et al. [18] found optimal solution for all the problem combinations in Table 8. The algorithm of GALA which was proposed by Yilmaz Eroglu et al. [21] also solved the problems on all these points in Table 8 with optimal values. For the job splitting situation the proposed MIP3 model which is introduced in Section 3.3, managed to find the optimal solutions for 5 combinations (6 jobs, 2 and 4 machines; 7 jobs, 2 and 4 machines; 8 jobs, 4 machines) in acceptable CPU time. The proposed algorithm of GAspLA could manage to find the optimal results in a couple of seconds on these points. MIP3 and GAspLA algorithms for job splitting situation find the same or better results for the combinations as compared with the nonjob splitting situation. In addition, the implementation of GAspLAMIP which is performed by using GAspLA results as initial solution for MIP3 is solving 9 combinations (6 jobs, 2 and 4 machines; 7 jobs, 2 and 4 machines; 8 jobs, 2 and 4 machines; 9 jobs, 2 and 4 machines; 10 jobs, 2 machines) optimally in acceptable CPU time. The elapsed time of the GAspLAMIP is less than MIP3 as it can be seen in Table 8. The optimal makespan values of GAspLAMIP are the same with the result of GAspLA for the studied combinations. The results for these combinations demonstrated that the makespan values of GAspLA are close to the lower bounds. In the next subsection the performance of the GAspLA for the other combinations are shown in detail.

### 6.3. The Results of GAspLA for Some Literature Problems

To test the proposed GAspLA algorithm, *SchedulingResearch* 2005 [4] dataset was used. ACO which was proposed by Arnaout et al. [19], GALA which was proposed by Yilmaz Eroglu et al. [21], and our new approach GAspLA were compared using the mentioned dataset.

There is no dataset for the problem of *R*_*m*_/*S*_*ijk*_/*C*_max⁡_ with job splitting in the literature according to our knowledge. An original dataset did not contain number of splits. For that reason, number of splitting has been randomly generated between 1 and 3. For all instances, the processing and setup times that were uniformly distributed between 50 and 100 are called balanced datasets. For small instances, the combinations were 2 machines and 6, 7, 8, 9, 10, and 11 jobs, 4 machines and 6, 7, 8, 9, 10, and 11 jobs, 6 machines and 8, 9, 10, and 11 jobs, and 8 machines and 10 and 11 jobs. For large instances, the combinations were 4 machines and 20 jobs, 6 machines and 20 and 40 jobs, 8 machines and 20 and 60 jobs, and 10 machines and 20 and 40 jobs. Each machine-job combination was tested with 15 instances of the problem and the average deviation from a lower bound (LB) was calculated. The method for calculation of LB was explained in the paper of Arnaout et al. [19]. And the same LB values are used.

The percent deviation from lower bound is calculated as follows

(30) $$\begin{array}{c} \text{Percent}\;\text{deviation}\;\text{from}\;\text{LB}=\frac{C_{\max_{\text{Algorithm}}}-\text{LB}}{\text{LB}}\cdot 100, \end{array}$$

where *C*_max⁡_Algorithm__ is the makespan value of the algorithm and LB is lower bound for the concerned instance.

**Figure 13:**
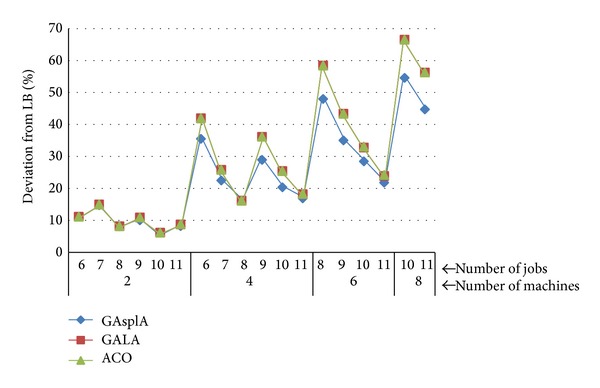
Deviation of each algorithm from the LB-small instances.

Figure 13 summarizes the relative deviation of each algorithm from LB for small problem combination. It's clear from Figure 13 that the GAspLA outperformed the others for the small problem sets. So if the problem can be split in subjobs, our new approach gives better results.

**Figure 14:**
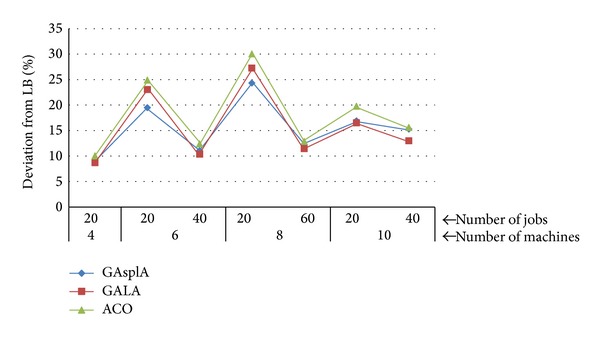
Deviation of each algorithm from the LB-large instances.

Figure 14 summarizes the relative deviation of each algorithm from LB for large problem combination. It's clear from Figure 14 that the GAspLA outperformed the ACO on each combination. The GAspLA outperformed the GALA on 6 machines and 20 jobs and 8 machines and 20 jobs combinations. The GAspLA finds similar results with GALA on the other points. The number of splitting for GAspLA can be selected between 1 and 3 as pointed out before. The GAspLA decides not to split, so the number of splitting is 1, in the case of not finding better results. This is the main reason of obtaining similar results with the GALA, which is the nonjob splitting version of this study.

## 7. Conclusions and Future Direction of Research

The present paper presented mixed integer programming (MIP) models, genetic-local search algorithm (GAspLA), and an implementation of GAspLAMIP for the unrelated parallel machine scheduling problem with machine-dependent and sequence-dependent setup times and job splitting property to minimize the makespan. In this problem, it was assumed that a job can be split into a number of subjobs and these subjobs are processed on the unrelated parallel machines independently. Very little research appears to have been undertaken on this general model. The proposed hybrid genetic-local search algorithms are coded in C# language according to proposed methods.

The first contribution of this paper is capability of the developed algorithms to make splitting and scheduling simultaneously with variable number of subjobs, where, to the best of our knowledge, no work has been published on an algorithm with these properties. The proposed hybrid genetic-local search algorithm (GAspLA) is innovated in its use of random key numbers to determine the number of subjobs for each job and managing them to sequence on the machines. In order to overcome the problem of managing random key numbers according to the desired job sequence that might appear during local search operation which implemented in the genetic algorithm, some methods are developed. The developed algorithms satisfy the adaptation of results of local search into the genetic algorithms with minimum relocation operation of genes' random key numbers. This is the second contribution of the paper. The third contribution of this paper is developed MIP models, which are coded in MPL and solved using CPLEX 11.0 solver. According to the best of our knowledge, there is no MIP approach for the problem type studied in this research. The results that are reported here indicate that the mentioned problem can be solved using the developed MIP models. Three mathematical models are presented on this topic. In the MIP1 and the MIP2, numbers of the subjobs will be determined by models between one and the maximum number of subjobs. In the MIP3, the numbers of subjobs will be determined randomly between one and maximum number of subjobs beforehand and will be given as an input data to the model. In the MIP1, the quantities of subjobs are not equal. In the MIP2 and MIP3 the quantities of subjobs are equal. The fourth contribution of this paper is an implementation of combining the results from GAspLA with the developed MIP formulation. This implementation (GAspLAMIP) also shows us the optimality of the results of GAspLA for the studied combinations. We used Design of Experiment to select the parameter values for hybrid genetic-local search algorithms. The proposed methods are tested on a set of small and large problems taken from the literature (*SchedulingResearch* 2005 [4]), and the computational results validate the effectiveness of the proposed algorithm.

The algorithms suggested in this paper may be useful for customer oriented market environments just like weaving process of textile industry that this study is originated from. And also drilling of printed circuit board (PCB) manufacturing and dicing of semiconductor wafer manufacturing have similar production environment.

There are balanced, setup times dominant and processing time dominant datasets in this *SchedulingResearch* 2005 [4] database. In this research we made the comparisons using balanced datasets. Running the algorithm using the remaining datasets might give interesting results. In this research, we are applying local search in each generation. Determining the conditions of applying local search may accelerate the algorithm. In order get closer to the real system, machine eligibility would be included into the algorithms. Generating hyperheuristics through the use of different metaheuristics' strengths may be a worthwhile avenue of research. Application of the proposed algorithm to multiobjective fitness functions would be a novel research topic. Other constraints, such as priority conditions, could be incorporated into future research. The other parallel machines problems like batch-processing machines seem also interesting.

## Figures and Tables

**Algorithm 1:**
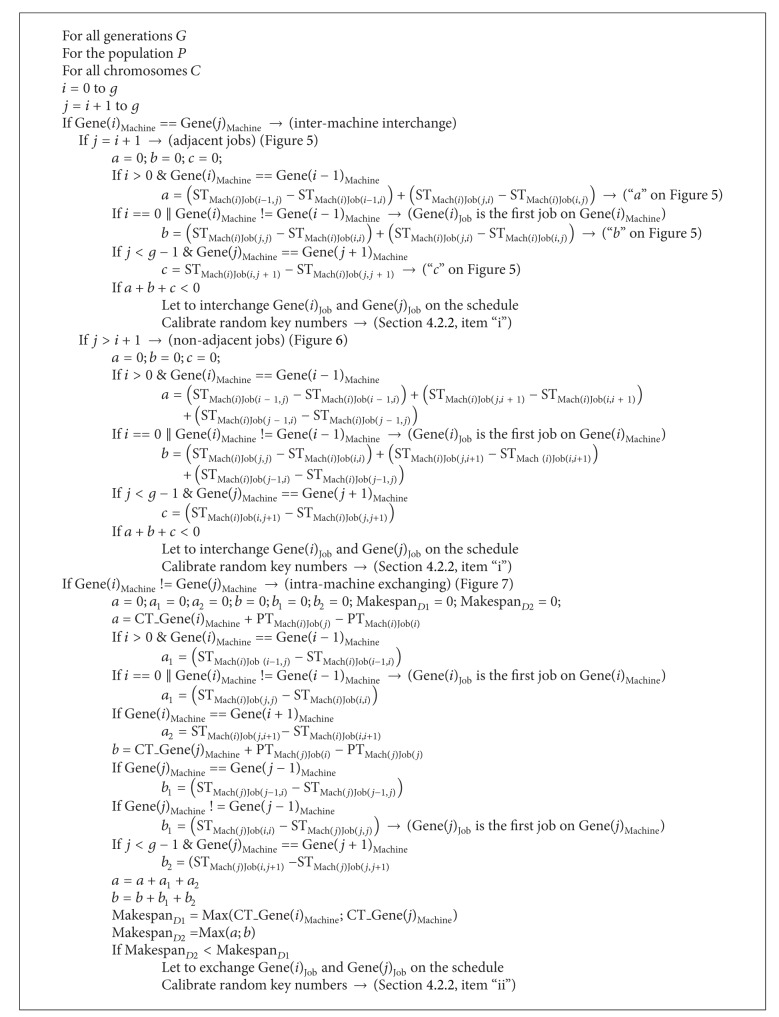
Local search.

**Table 1:** Jobs' total processing times on machines.

*P*_*ij*_ *P*_*ij*_	Job_1_	Job_2_	Job_3_
Machine_1_	90	60	30
Machine_2_	95	70	40

**Table 2:** Setup times for Machine_1_.

*S*_*ijk*_ *S*_*ijk*_	Job_1_	Job_2_	Job_3_
Job_1_	—	8	6
Job_2_	2	—	4
Job_3_	3	7	—

**Table 3:** Setup times for Machine_2_.

*S*_*ijk*_ *S*_*ijk*_	Job_1_	Job_2_	Job_3_
Job_1_	—	2	6
Job_2_	4	—	3
Job_3_	2	1	—

**Table 4:** Sample gene structure for an example that contains seven jobs and two machines.

Gene number	0	1	2	3	4	5	6
Machine number	0	0	0	0	1	1	1
Job number	3	4	6	1	0	2	5

**Table 5:** Initial values for the binary variable set.

Machine number *i *	Subjob sequence				Value of binary variable *X*_*ijrks*_
	*j *	*r *	*k *	*s *	
1	0	1	3	1	1
2	0	1	1	1	1
2	1	1	4	1	1
3	0	1	6	1	1
3	6	1	5	1	1
4	0	1	2	1	1
4	2	1	6	2	1

**Table 6:** Parameter values used to assess the proposed genetic algorithm.

Parameter	Value
Population size (*P*_size_)	80; 100
Probability of crossover (*P*_*c*_)	0.5; 1.0
Probability of mutation (*P*_*m*_)	0.2; 0.5

**Table 7:** Parameter estimates.

Term	Effect	Coef.	SE coef.	*T *	*P *
Constant		606.025	0.2789	2172.53	0.000
*P*_size_	−0.450	−0.225	0.2789	−0.81	0.426
*P*_*c*_	−2.450	−1.225	0.2789	−4.39	**0.000**
*P*_*m*_	1.350	0.675	0.2789	2.42	**0.021**
*P*_size_∗*P*_*c*_	0.050	0.025	0.2789	0.09	0.929
*P*_size_∗*P*_*m*_	−0.150	−0.075	0.2789	−0.27	0.790
*P*_*c*_∗*P*_*m*_	−1.150	−0.575	0.2789	−2.06	**0.047**
*P*_size_∗*P*_*c*_∗*P*_*m*_	0.750	0.375	0.2789	1.34	0.188

**Table 8:** The comparison of solution methods.

# of Job	# of Machine	Non job splitting situation				Job splitting situation					
		MIP0		GALA		MIP3		GAspLA		GAspLAMIP	
		Makespan Value	Elapsed Time (second)	Makespan Value	Elapsed Time (second)	Makespan Value	Elapsed Time (second)	Makespan Value	Elapsed Time (second)	Makespan Value	Elapsed Time (second)
6	2	390	3	390	3	390	545	390	9	390	14
6	4	235	3	235	4	234	1106	234	13	234	16
7	2	484	23	484	4	470.5	35172	470.5	13	470.5	204
7	4	258	3	258	5	245	7200	245	22	245	26
8	2	494	232	494	5	—	>86400	494	16	494	245
8	4	264	6	264	7	264	80096	264	27	264	29
9	2	627	2120	627	6	—	>86400	627	19	627	45394
9	4	345	6345	345	9	—	>86400	331	30	331	38376
10	2	647	28144	647	7	—	>86400	647	28	647	29842
10	4	360	58626	360	11	—	>86400	352.5	40	—	>86400

## Appendix

Here we give pseudocode for the local search. For brevity only the key steps are detailed.

C# notation is used.

See Algorithm 1.
